# Supplementary material for: Contrasting Global and Patient‐Specific Regression Models via a Neural Network Representation
Source: Biom J. 2026 Mar 23;68(2):e70126. doi: 10.1002/bimj.70126 (PMC13010063; doi:10.1002/bimj.70126)

CompositeAE Comp. Space with Subgroup Highlighting (_seed513_train_only_

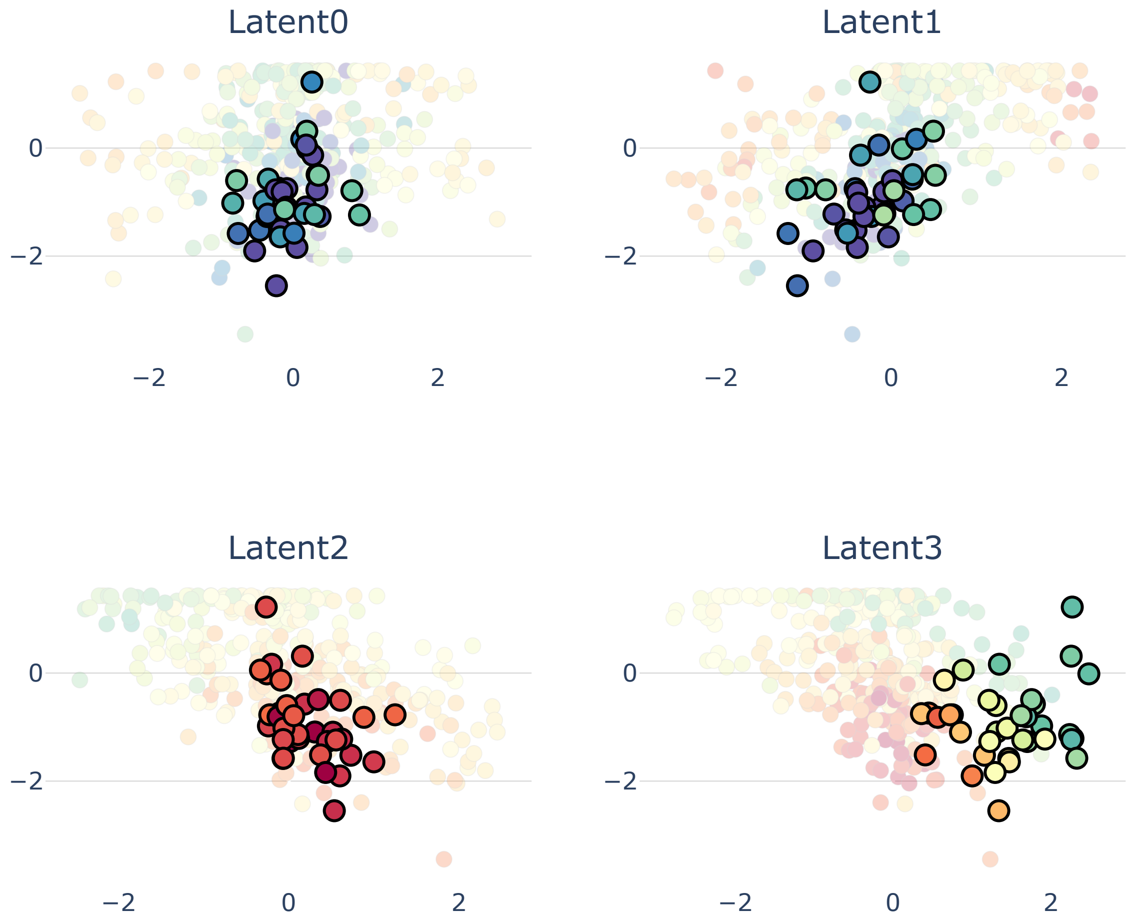

Supplement: Supplementary file 1 — Supporting Information [file BIMJ-68-e70126-s001.zip › AEnabledLoReg-main/results/figures/Figure3or4_A_latent_subgroup_highlight_CompositeAE_seed513_train_only_Dynamic_Latent2_below_LCI.pdf]
