# Supplementary figures and images for: Contrasting Global and Patient‐Specific Regression Models via a Neural Network Representation
Source: Biom J. 2026 Mar 23;68(2):e70126. doi: 10.1002/bimj.70126 (PMC13010063; doi:10.1002/bimj.70126)

# CompositeAE Comp. vs Outcome (\_seed513\_train\_only\_Dynamic\_Latent0\_ab

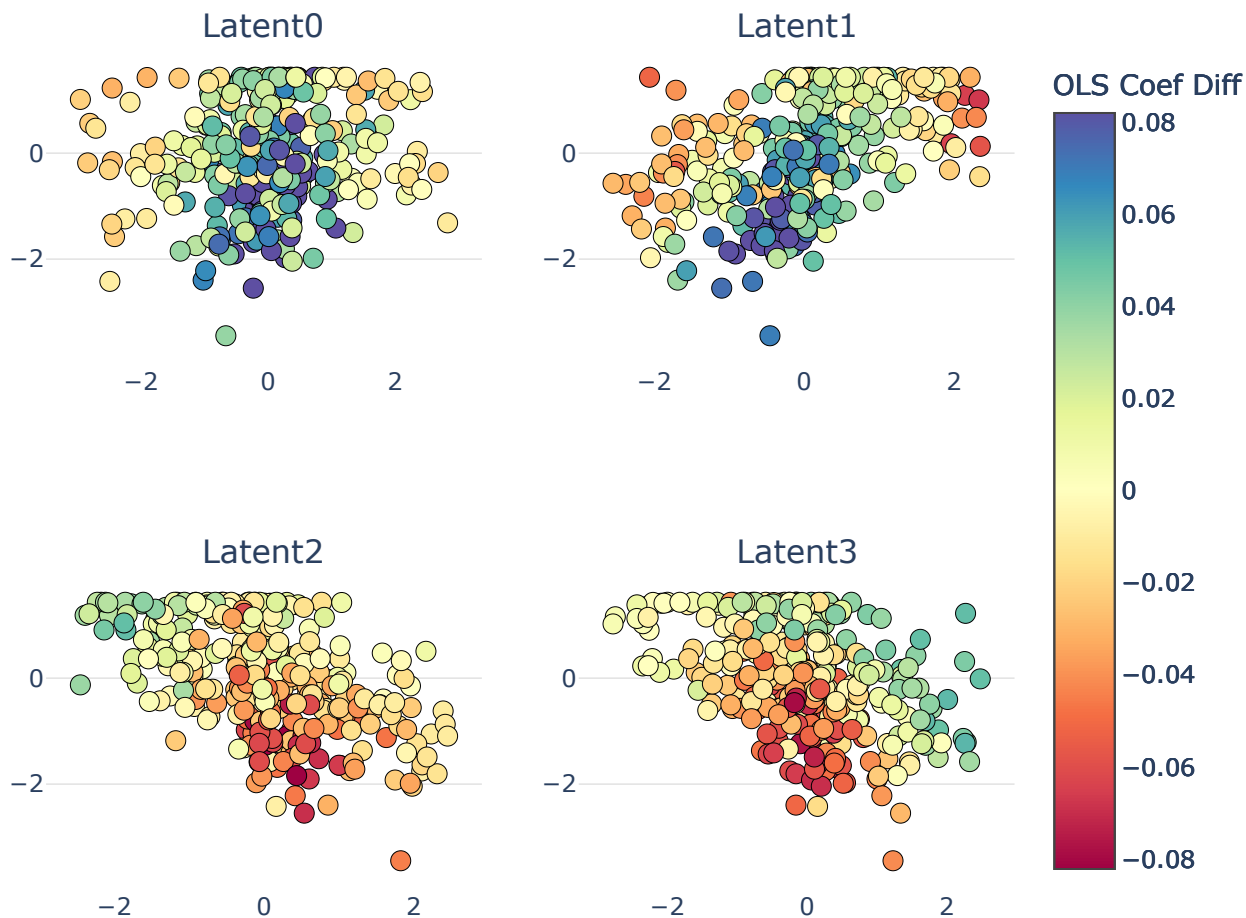

Supplement: Supplementary file 1 — Supporting Information [file BIMJ-68-e70126-s001.zip › AEnabledLoReg-main/results/figures/Figure2_latent_coeffs_CompositeAE_seed513_train_only_Dynamic_Latent0_above_UCI.pdf]

# CompositeAE Comp. vs Outcome (\_seed513\_train\_only\_Dynamic\_Latent1\_ab

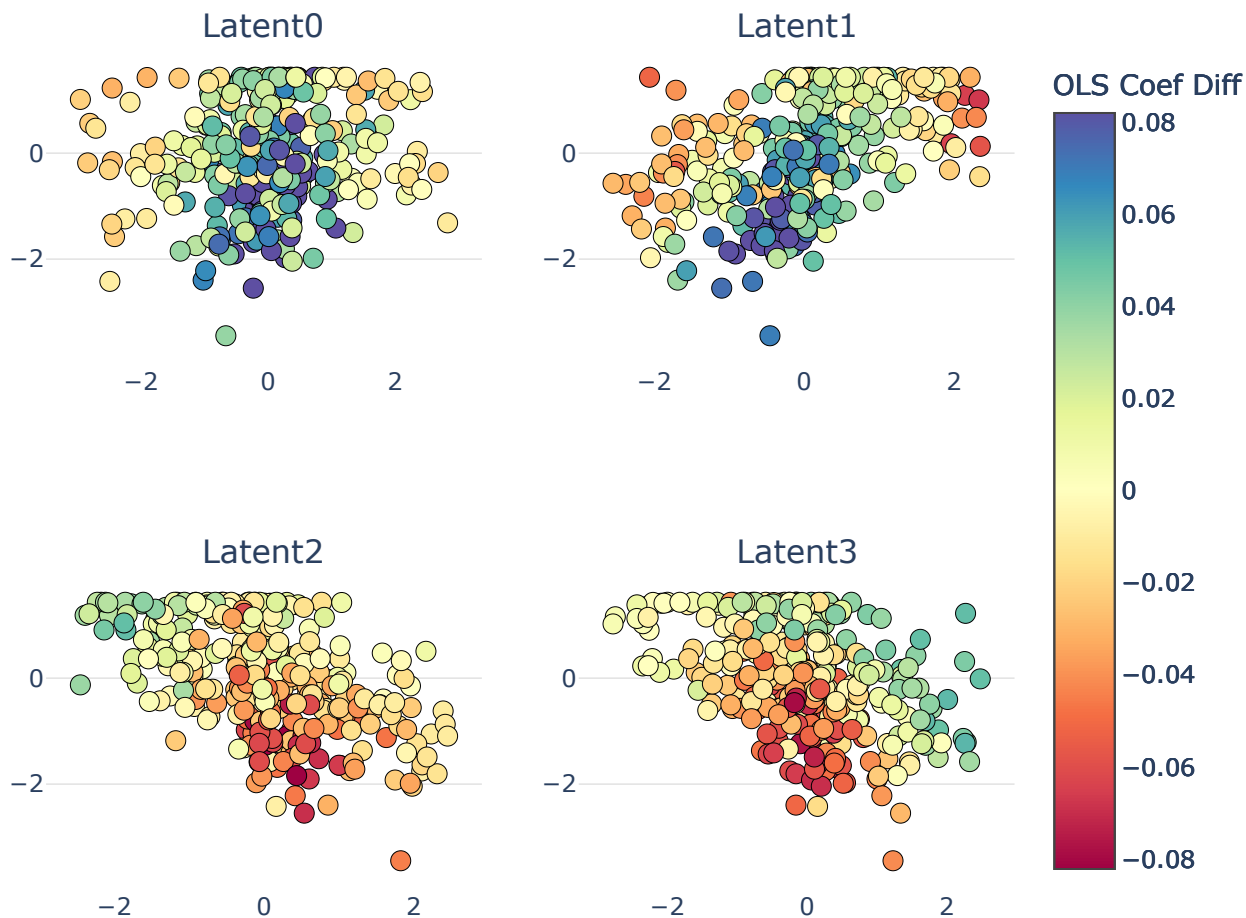

Supplement: Supplementary file 1 — Supporting Information [file BIMJ-68-e70126-s001.zip › AEnabledLoReg-main/results/figures/Figure2_latent_coeffs_CompositeAE_seed513_train_only_Dynamic_Latent1_above_UCI.pdf]

# CompositeAE Comp. vs Outcome (\_seed513\_train\_only\_Dynamic\_Latent2\_be

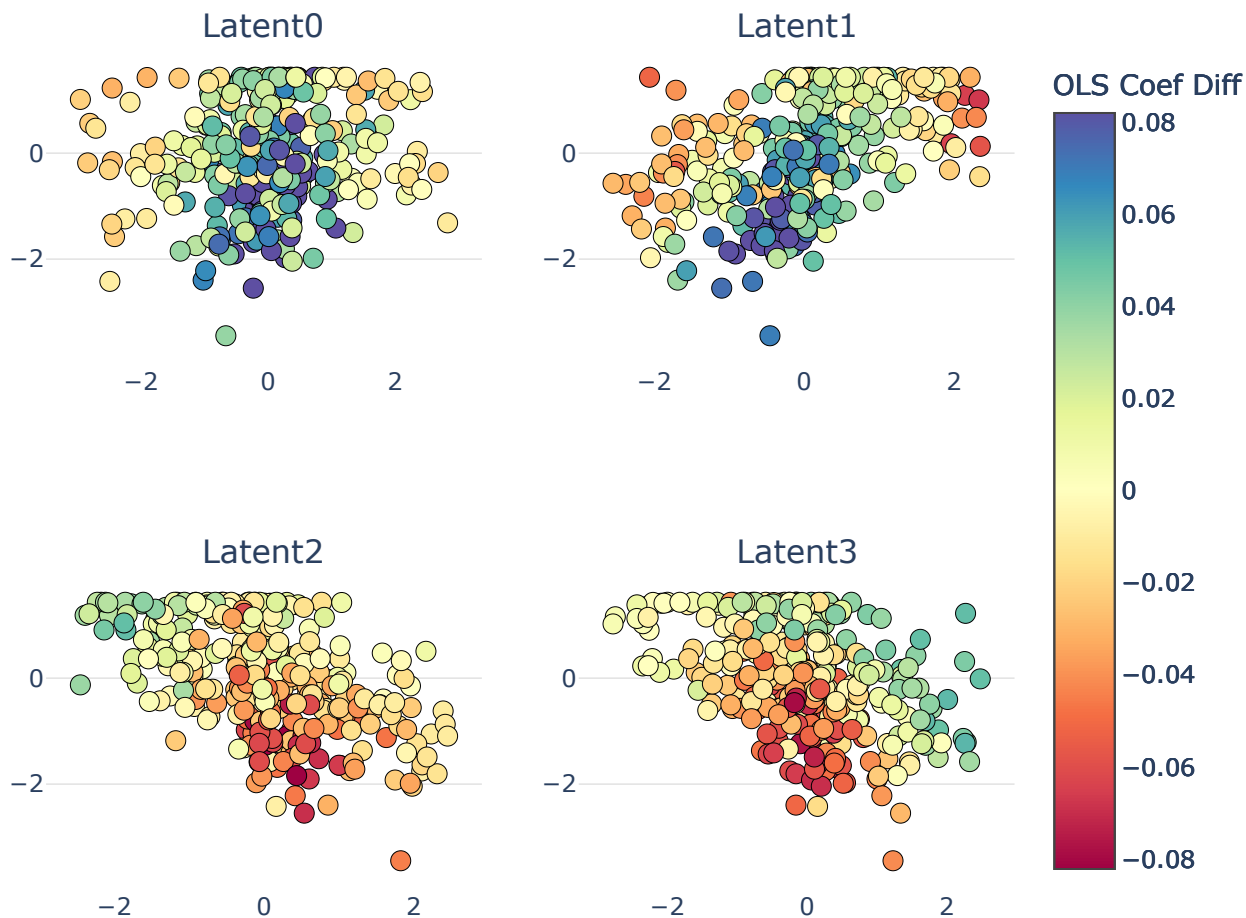

Supplement: Supplementary file 1 — Supporting Information [file BIMJ-68-e70126-s001.zip › AEnabledLoReg-main/results/figures/Figure2_latent_coeffs_CompositeAE_seed513_train_only_Dynamic_Latent2_below_LCI.pdf]

# CompositeAE Comp. vs Outcome (\_seed513\_train\_only\_Dynamic\_Latent3\_be

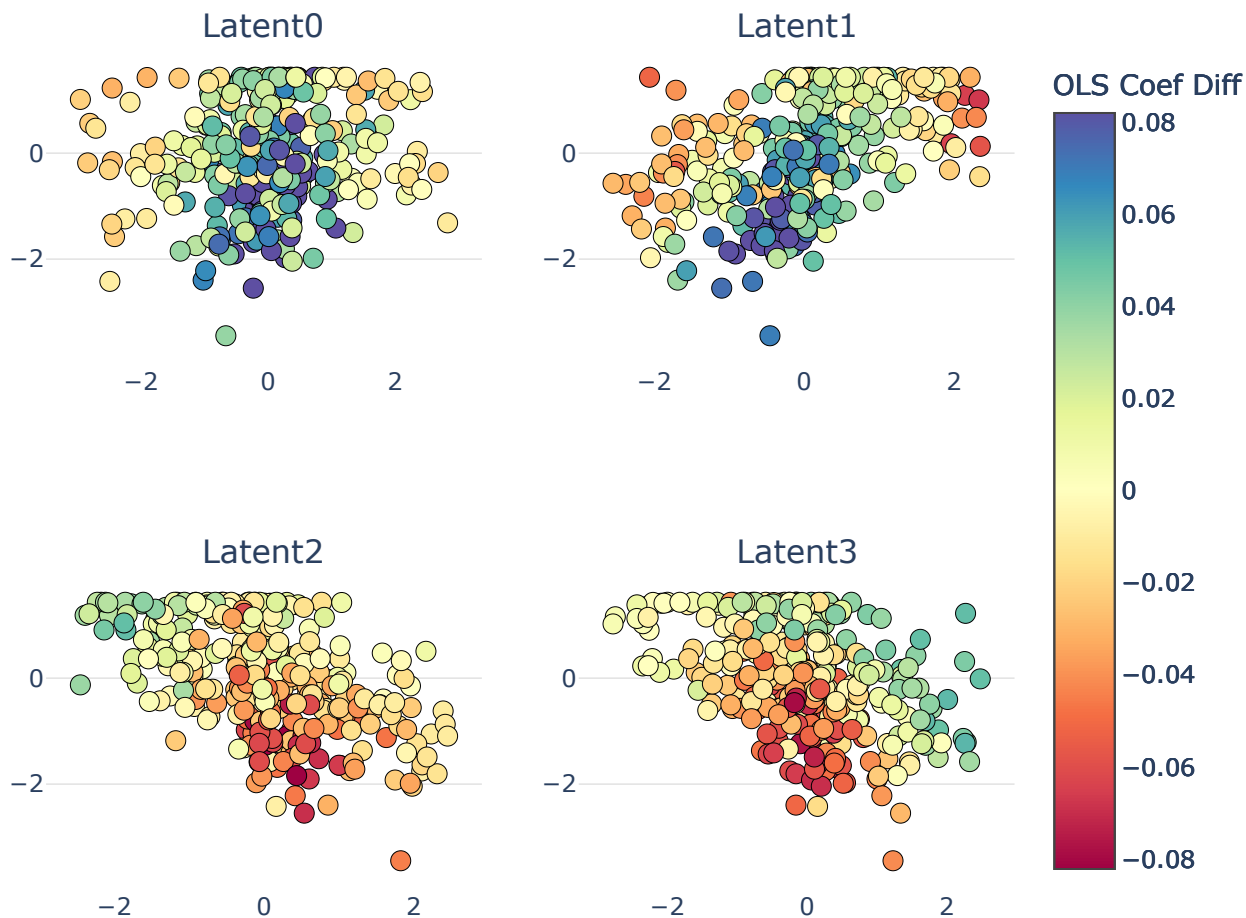

Supplement: Supplementary file 1 — Supporting Information [file BIMJ-68-e70126-s001.zip › AEnabledLoReg-main/results/figures/Figure2_latent_coeffs_CompositeAE_seed513_train_only_Dynamic_Latent3_below_LCI.pdf]

# Original Feature Effects by Subgroup (CompositeAE - \_\_seed513\_train\_only\_D

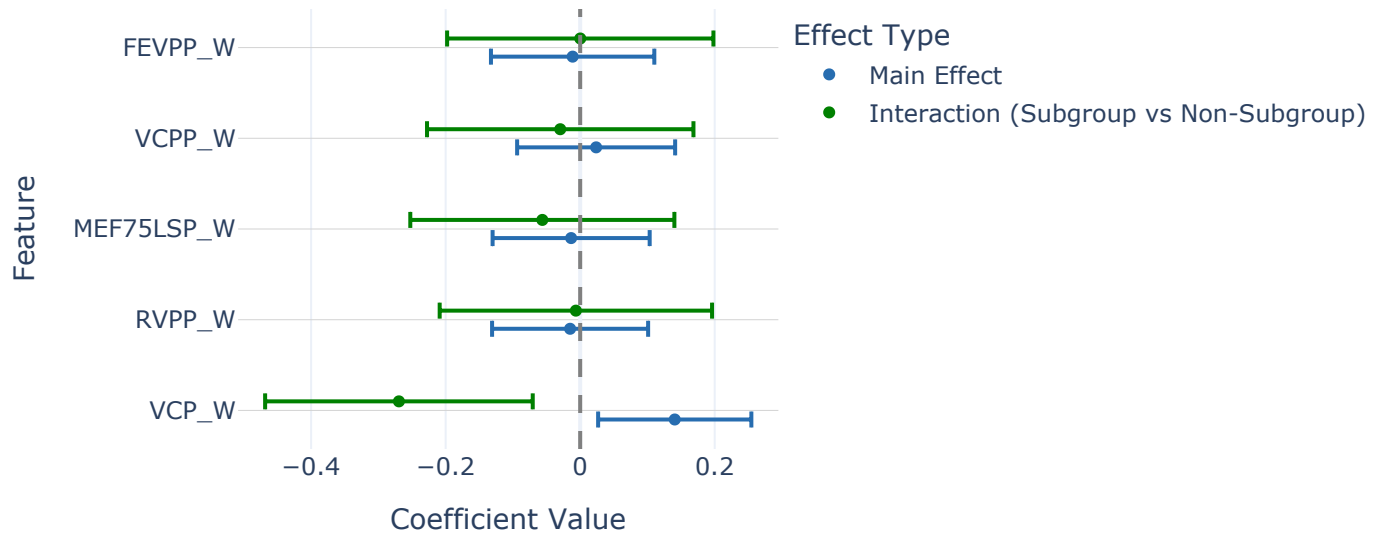

Supplement: Supplementary file 1 — Supporting Information [file BIMJ-68-e70126-s001.zip › AEnabledLoReg-main/results/figures/Figure3or4_B_forest_plot_original_features_CompositeAE_seed513_train_only_Dynamic_Latent0_above_UCI.pdf]

# Original Feature Effects by Subgroup (CompositeAE - \_seed513\_train\_only\_D

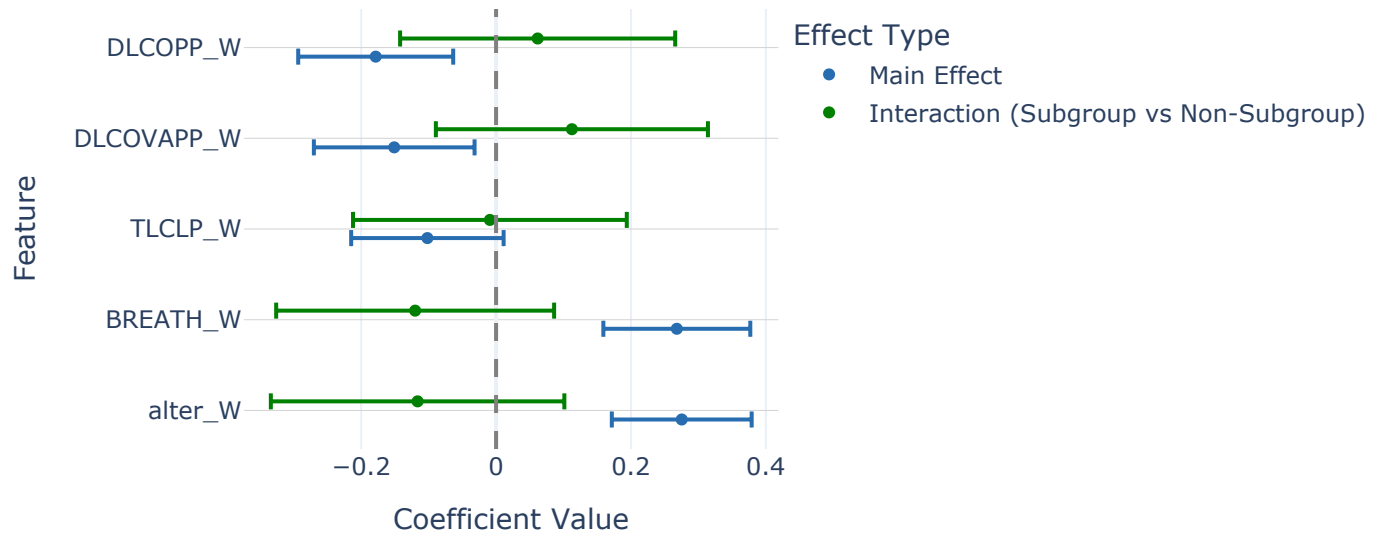

Supplement: Supplementary file 1 — Supporting Information [file BIMJ-68-e70126-s001.zip › AEnabledLoReg-main/results/figures/Figure3or4_B_forest_plot_original_features_CompositeAE_seed513_train_only_Dynamic_Latent1_above_UCI.pdf]

# Original Feature Effects by Subgroup (CompositeAE - \_seed513\_train\_only\_D

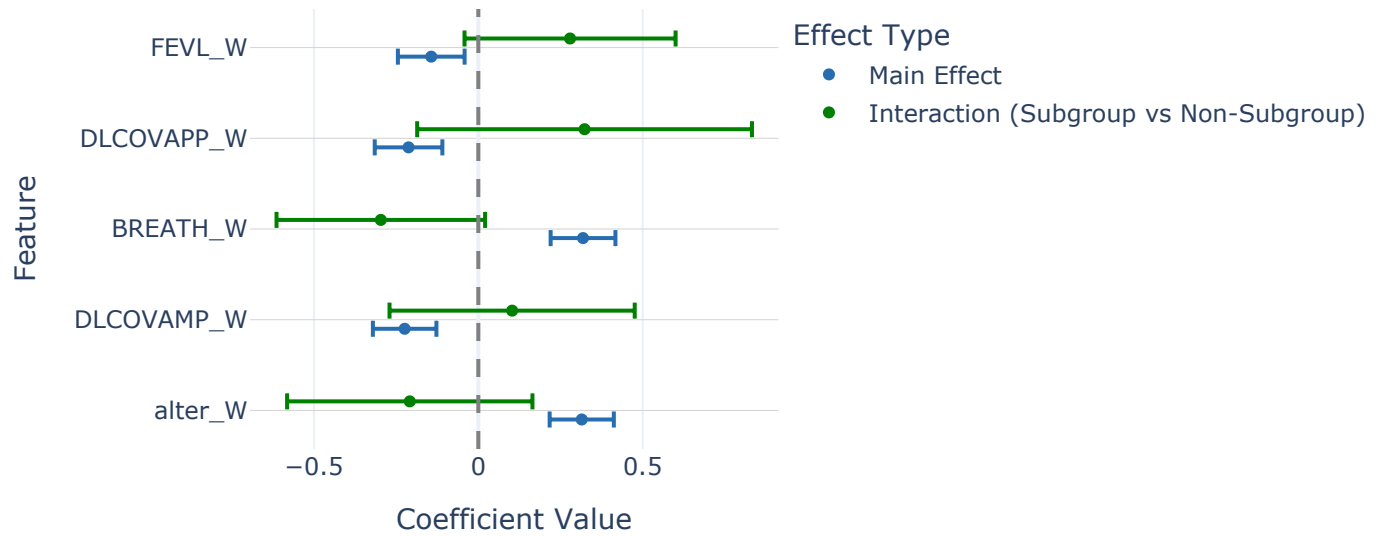

Supplement: Supplementary file 1 — Supporting Information [file BIMJ-68-e70126-s001.zip › AEnabledLoReg-main/results/figures/Figure3or4_B_forest_plot_original_features_CompositeAE_seed513_train_only_Dynamic_Latent2_below_LCI.pdf]

# Original Feature Effects by Subgroup (CompositeAE - \_\_seed513\_train\_only\_D

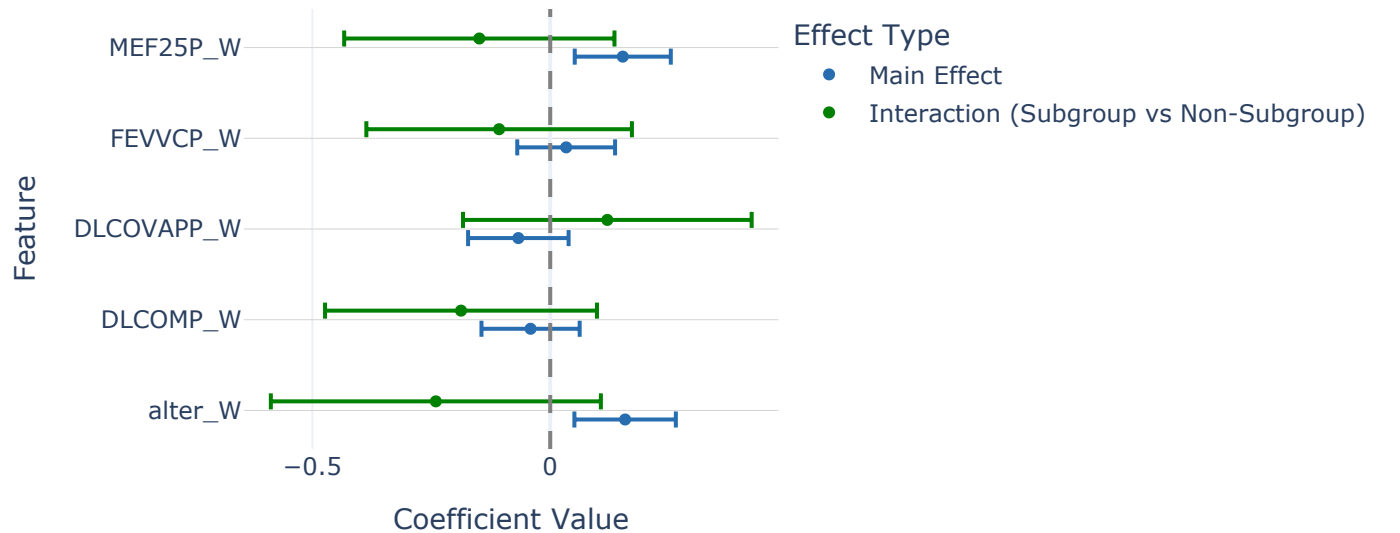

Supplement: Supplementary file 1 — Supporting Information [file BIMJ-68-e70126-s001.zip › AEnabledLoReg-main/results/figures/Figure3or4_B_forest_plot_original_features_CompositeAE_seed513_train_only_Dynamic_Latent3_below_LCI.pdf]

Z-Score Diff (Subgroup Train vs Pop Train, CompositeAE - \_seed513\_train\_on

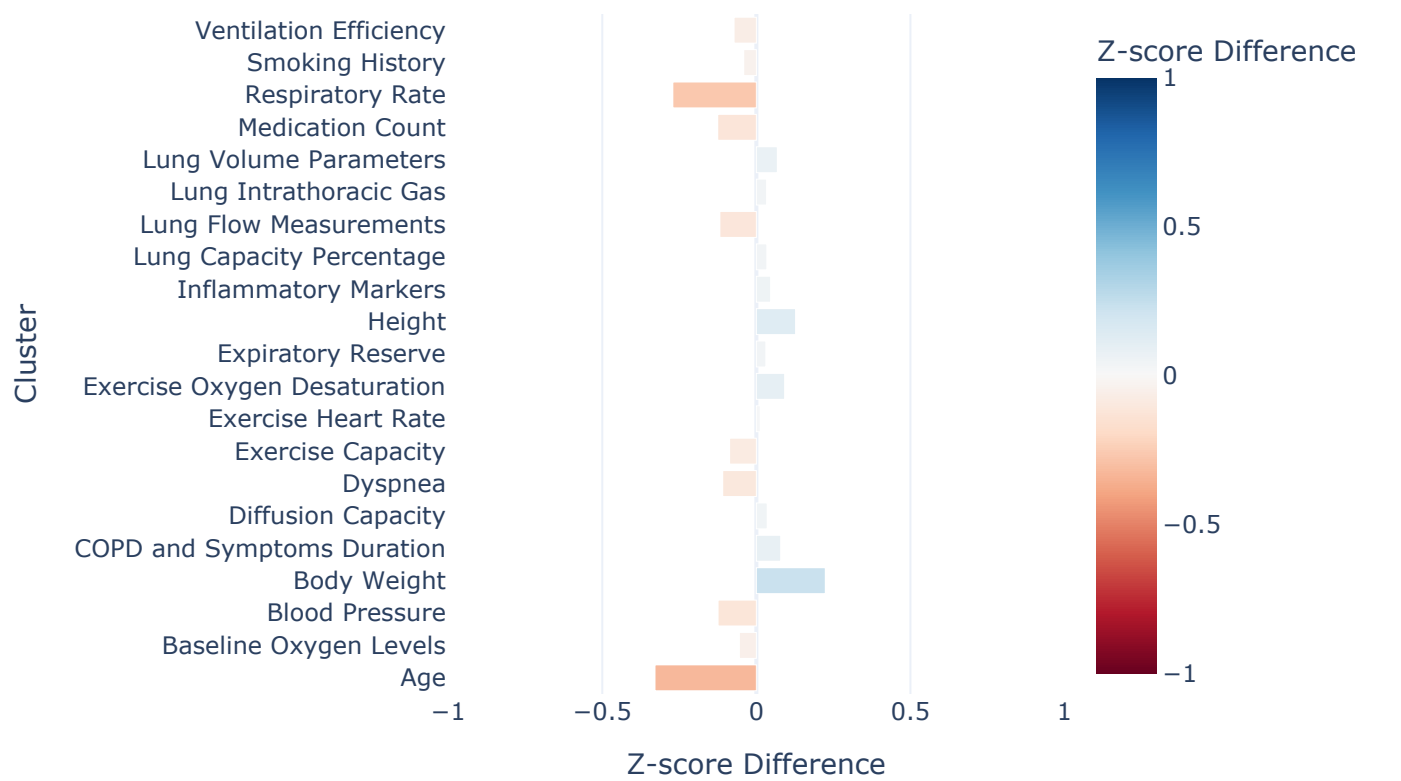

Supplement: Supplementary file 1 — Supporting Information [file BIMJ-68-e70126-s001.zip › AEnabledLoReg-main/results/figures/Figure3or4_C_z_profile_CompositeAE_seed513_train_only_Dynamic_Latent0_above_UCI.pdf]

Z-Score Diff (Subgroup Train vs Pop Train, CompositeAE - \_seed513\_train\_on

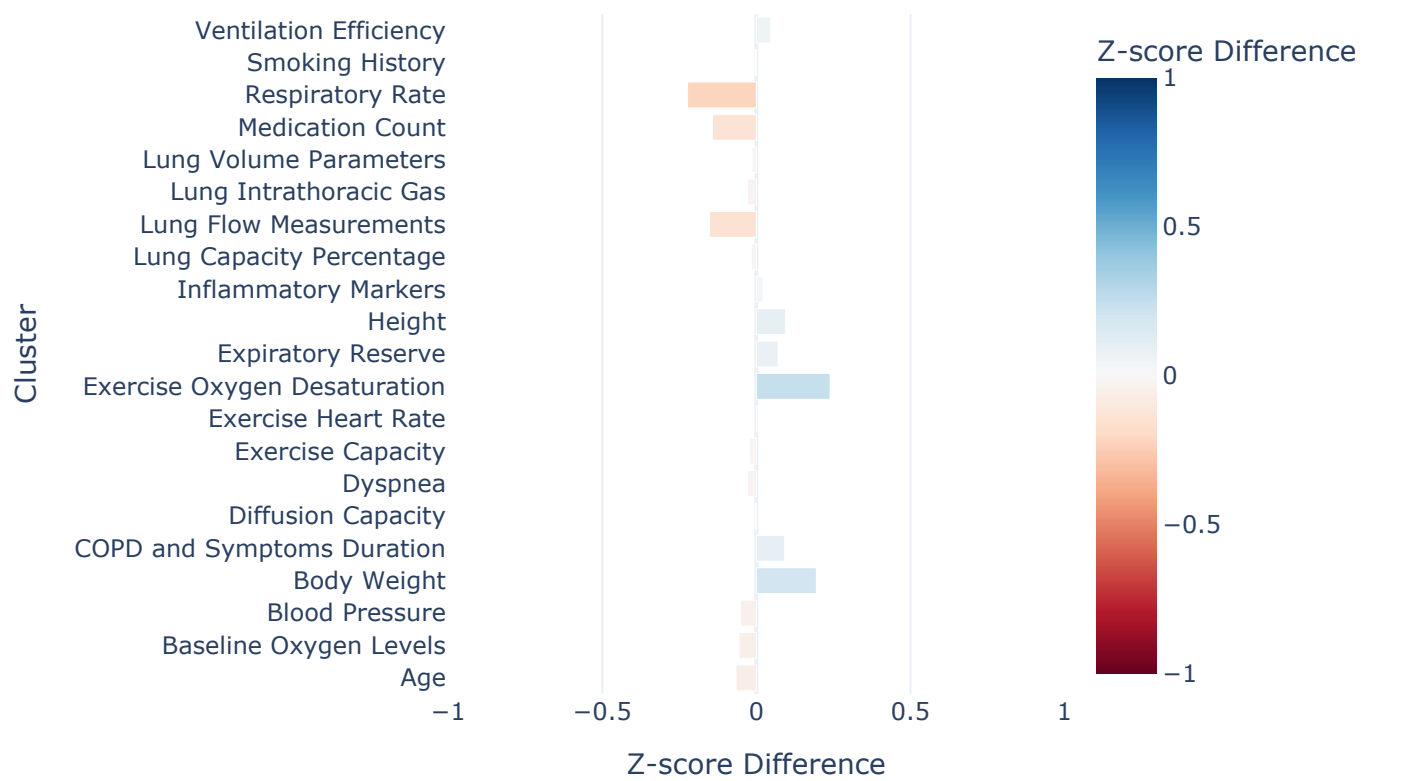

Supplement: Supplementary file 1 — Supporting Information [file BIMJ-68-e70126-s001.zip › AEnabledLoReg-main/results/figures/Figure3or4_C_z_profile_CompositeAE_seed513_train_only_Dynamic_Latent1_above_UCI.pdf]

Z-Score Diff (Subgroup Train vs Pop Train, CompositeAE - \_seed513\_train\_on

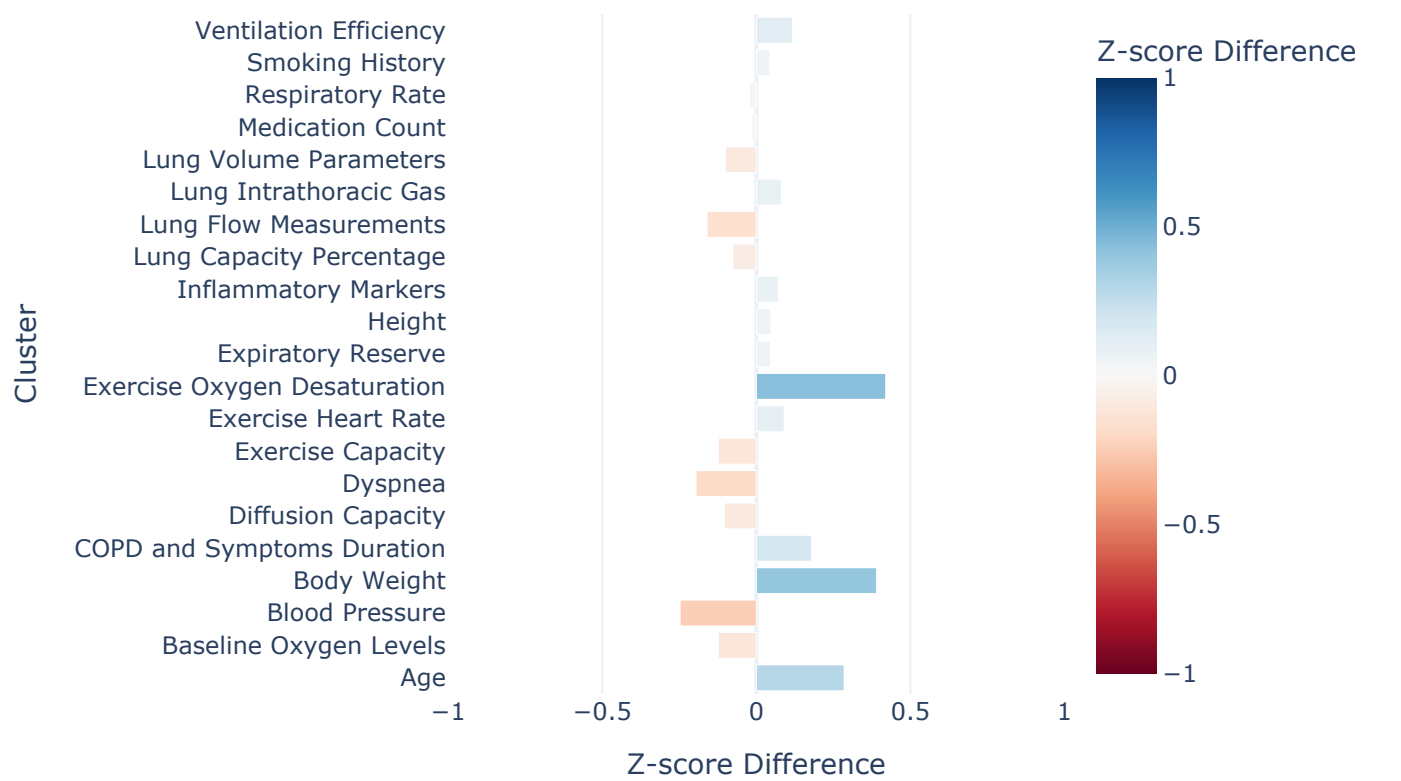

Supplement: Supplementary file 1 — Supporting Information [file BIMJ-68-e70126-s001.zip › AEnabledLoReg-main/results/figures/Figure3or4_C_z_profile_CompositeAE_seed513_train_only_Dynamic_Latent2_below_LCI.pdf]

Z-Score Diff (Subgroup Train vs Pop Train, CompositeAE - \_seed513\_train\_or

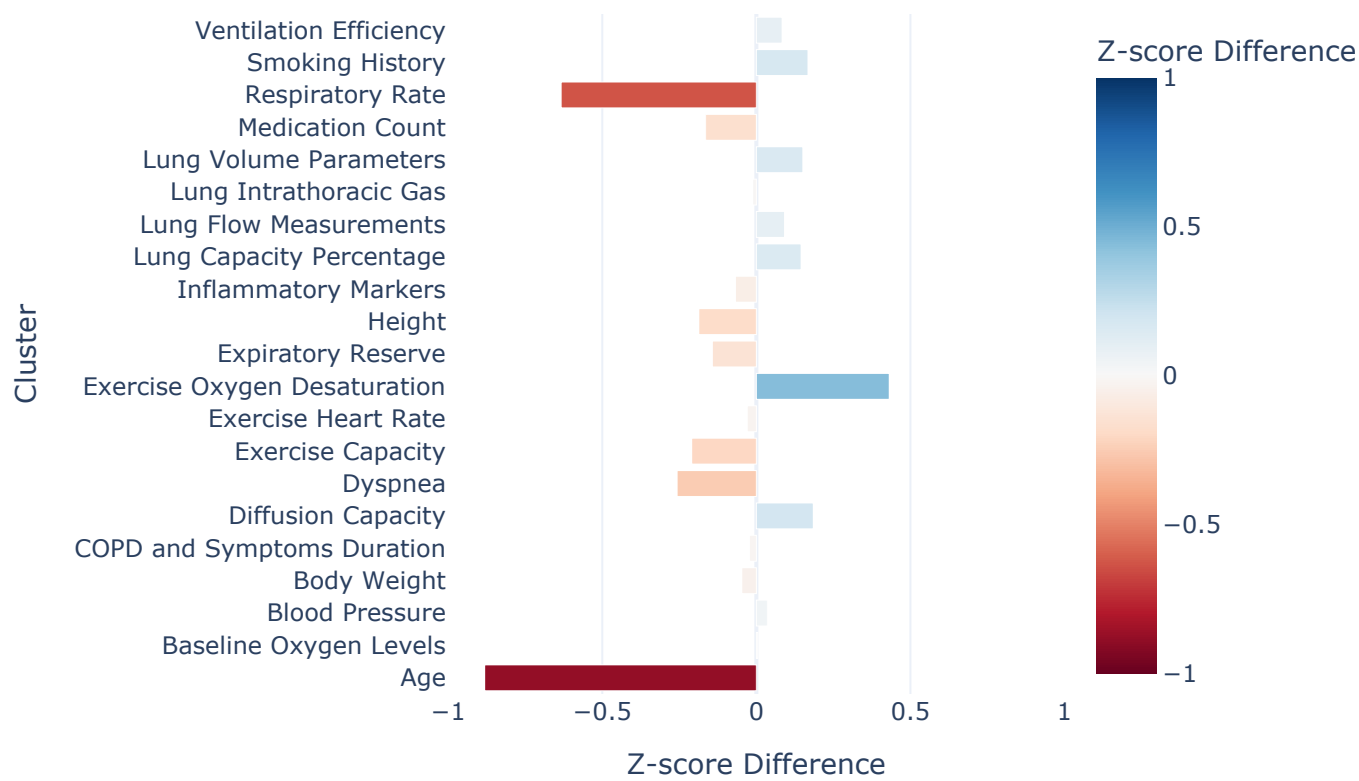

Supplement: Supplementary file 1 — Supporting Information [file BIMJ-68-e70126-s001.zip › AEnabledLoReg-main/results/figures/Figure3or4_C_z_profile_CompositeAE_seed513_train_only_Dynamic_Latent3_below_LCI.pdf]

# Original Feature Effects by Subgroup (CompositeAE - \_seed513\_combined\_Dy

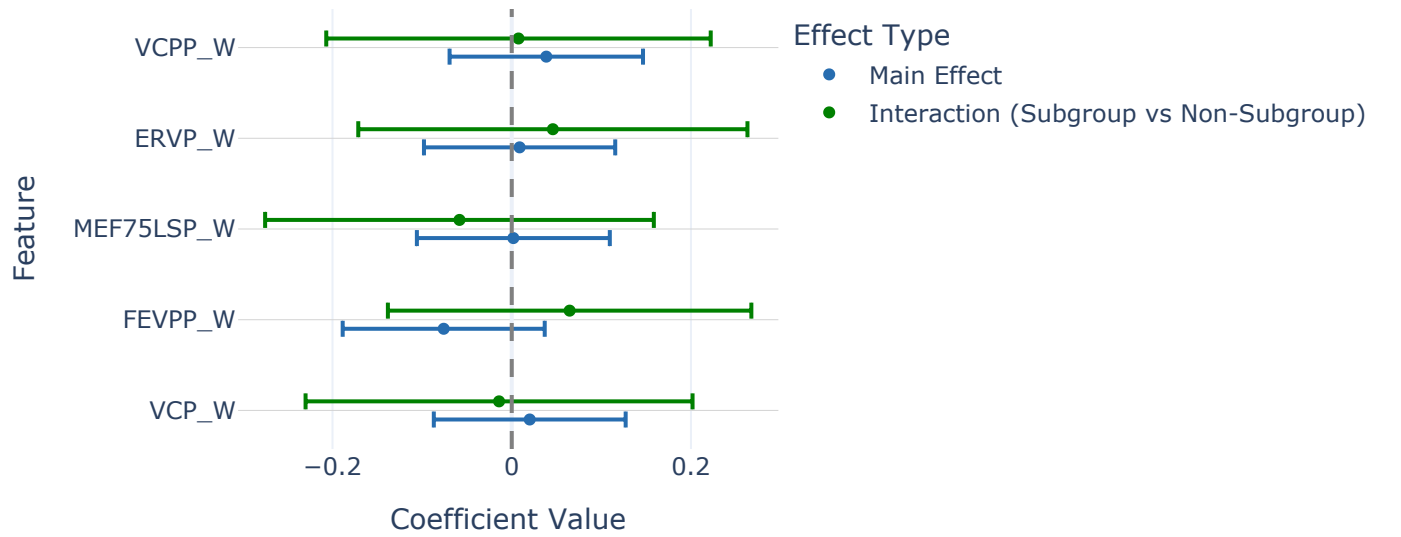

Supplement: Supplementary file 1 — Supporting Information [file BIMJ-68-e70126-s001.zip › AEnabledLoReg-main/results/figures/Figure5_forest_plot_original_features_CompositeAE_test__seed513_combined_Dynamic_Latent0_above_UCI.pdf]

## Original Feature Effects by Subgroup (CompositeAE - \_seed513\_combined\_Dy

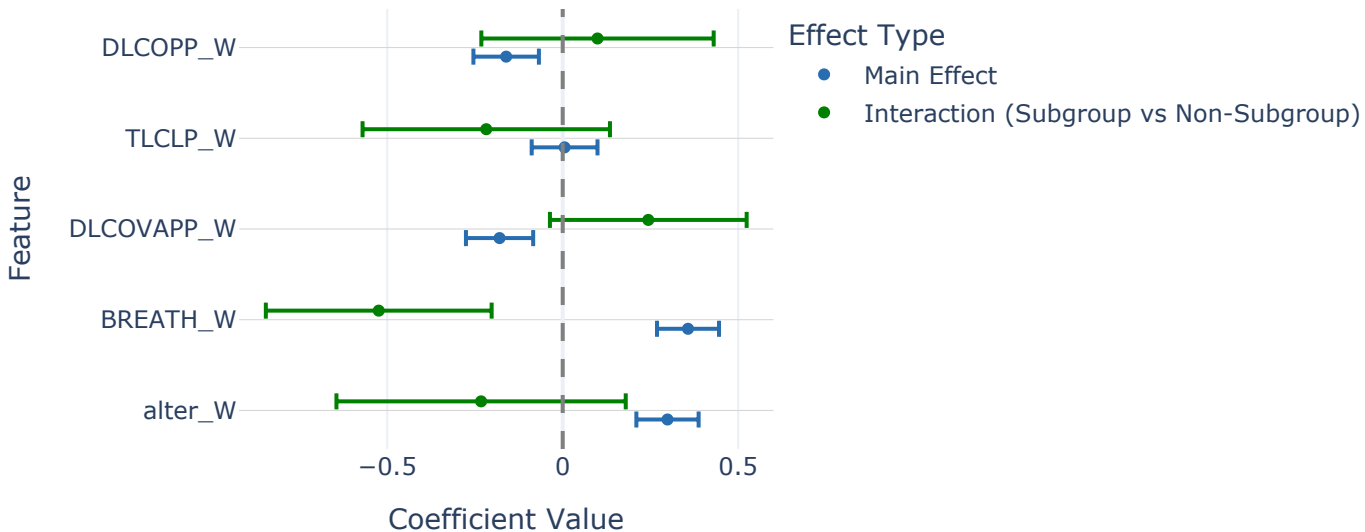

Supplement: Supplementary file 1 — Supporting Information [file BIMJ-68-e70126-s001.zip › AEnabledLoReg-main/results/figures/Figure5_forest_plot_original_features_CompositeAE_test__seed513_combined_Dynamic_Latent1_above_UCI.pdf]

Z-Score Diff (Subgroup Train vs Pop Train, CompositeAE - \_seed513\_combine

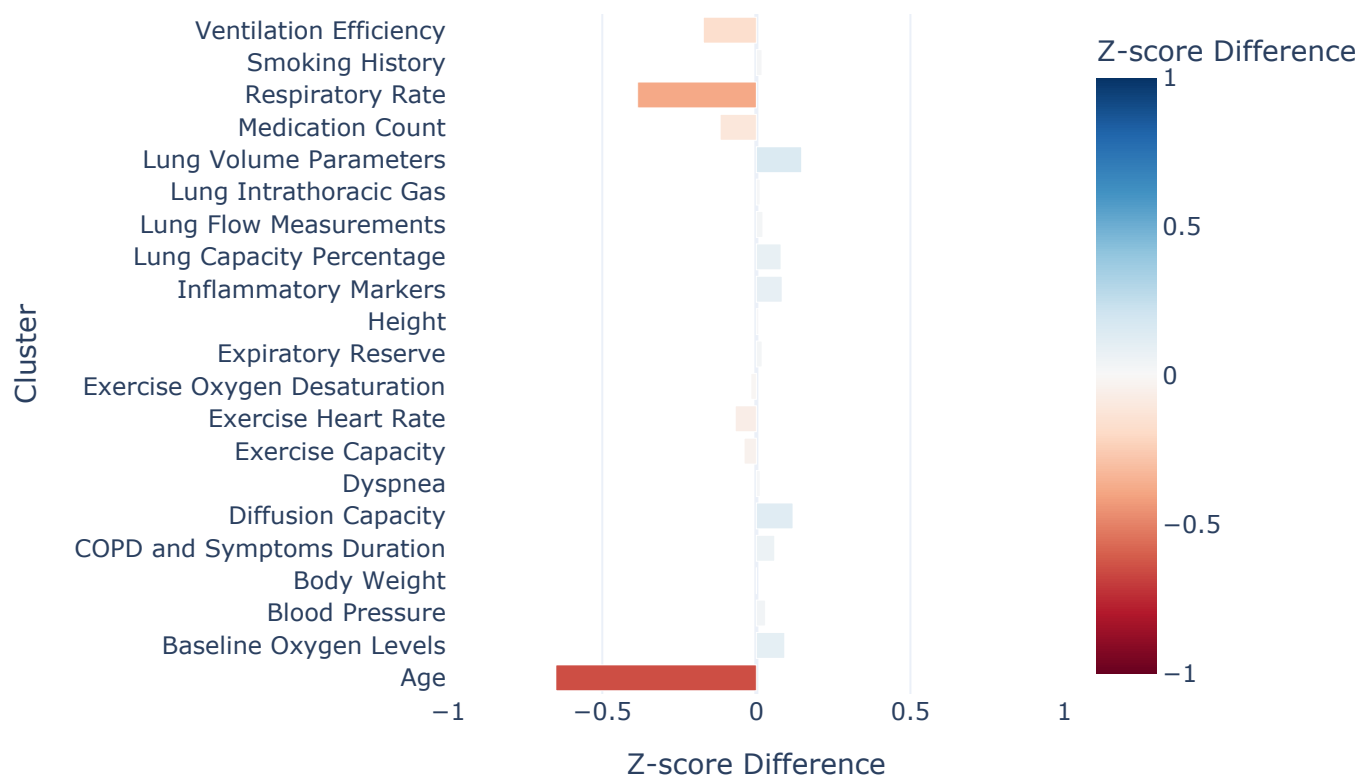

Supplement: Supplementary file 1 — Supporting Information [file BIMJ-68-e70126-s001.zip › AEnabledLoReg-main/results/figures/Figure5_z_profile_CompositeAE_seed513_combined_Dynamic_Latent0_above_UCI.pdf]

Z-Score Diff (Subgroup Train vs Pop Train, CompositeAE - \_seed513\_combine

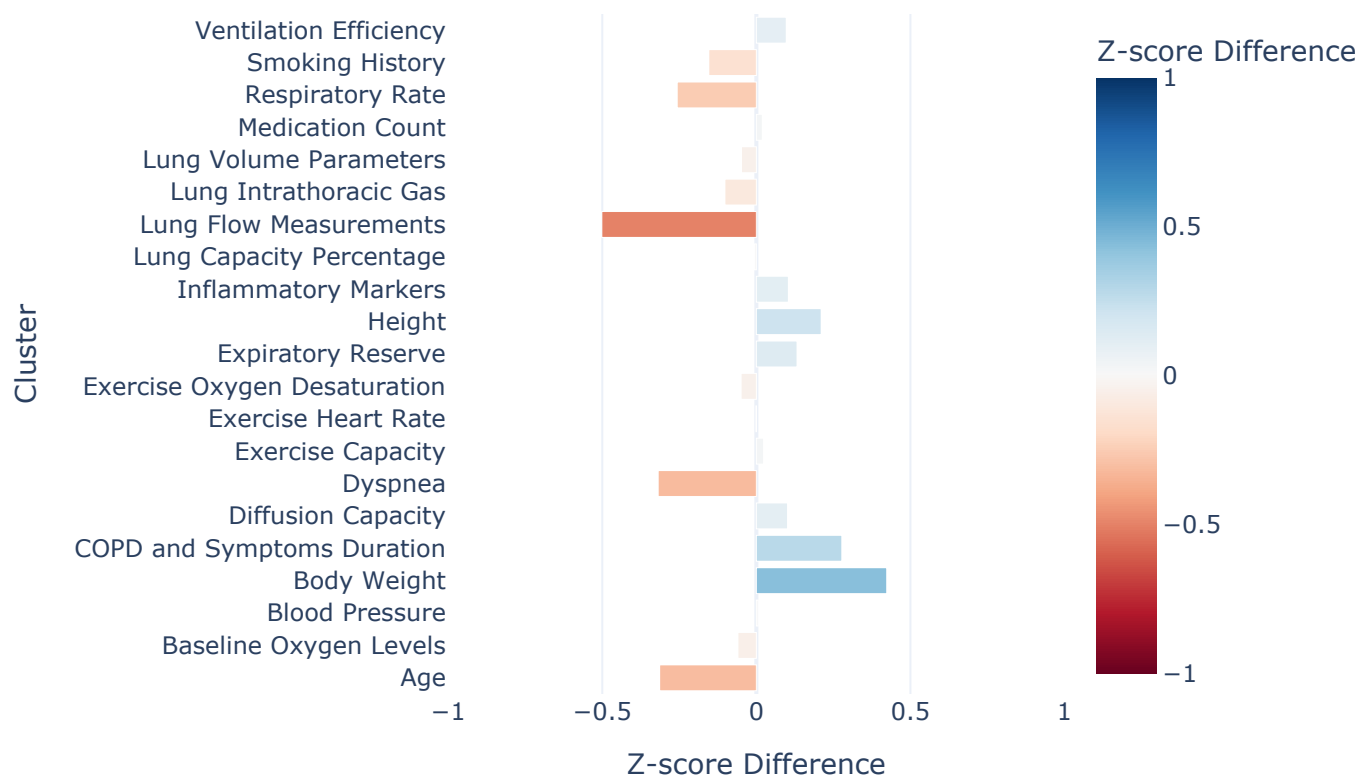

Supplement: Supplementary file 1 — Supporting Information [file BIMJ-68-e70126-s001.zip › AEnabledLoReg-main/results/figures/Figure5_z_profile_CompositeAE_seed513_combined_Dynamic_Latent1_above_UCI.pdf]
